# Genome Sequence of Colistin-Resistant Bacteremic *Shewanella algae* Carrying the Beta-Lactamase Gene *bla*_OXA-55_

**DOI:** 10.1155/2019/3840563

**Published:** 2019-06-10

**Authors:** Ying-Ju Chen, Kwong-Chung Tung, Yu-Kai Hong, Shi-Yu Chen, Yao-Ting Huang, Po-Yu Liu

**Affiliations:** ^1^Department of Food and Nutrition, Providence University, Taichung 43301, Taiwan; ^2^Department of Veterinary Medicine, National Chung Hsing University, Taichung 40227, Taiwan; ^3^Department of Computer Science and Information Engineering, National Chung Cheng University, Chiayi 62102, Taiwan; ^4^Division of Infectious Diseases, Department of Internal Medicine, Taichung Veterans General Hospital, Taichung 40705, Taiwan; ^5^Rong Hsing Research Center for Translational Medicine, National Chung Hsing University, Taichung 40227, Taiwan; ^6^Ph.D. Program in Translational Medicine, National Chung Hsing University, Taichung 40227, Taiwan

## Abstract

*Shewanella algae* is an emerging pathogen widely distributed in aquatic environment. Bacteremia is a major manifestation of *S. algae* infections, and there are increasing reports of antibiotic-resistant strains. However, little is known about the genomic characteristics of human bacteremic *S. algae*. Here, we report the results of the whole-genome sequencing of colistin-resistant *S. algae* TYL, a blood isolate. Chromosome-encoded *pmrC* associated with colistin resistance and *bla*_OXA-55_ gene intrinsic to *S. algae* was identified. Continuous surveillance for the emergence of *S*. *algae* is needed.

## 1. Introduction

*Shewanella algae* is a nonfermenting Gram-negative bacterium and autochthonous inhabitant of aquatic environments [1]. The organism could tolerate a wide range of physiological conditions [2] and has been documented as an emerging zoonotic pathogen [3]. It has been reported to cause ulcerative disease in marine fish [4] and shellfish [5]. The clinical spectrum in human infection is broad, including bacteremia, biliary tract infection, pneumonia, and soft tissue infections [6]. Bacteremia is one of the most common presentations of *S. algae* infections, which is associated with considerable morbidity and mortality [7].

The emergence of colistin resistance has become a major public heath challenge. The major advances of sequencing technology in past decades have enabled improved understanding of the genomic background of many important pathogens and resistance determinants. High-quality genomic data have become critical for the study of pathogenesis and therapeutic intervention of infectious diseases.

Although there are increasing reports of colistin-resistant *S. algae* bloodstream infections worldwide [8], little is known about the genomic characteristics of human bacteremic *S. algae*. Moreover, the widespread environmental nature of *S. algae* raises concern for its role as a resistance reservoir. In this study, we determined the whole-genome sequence of a colistin-resistant *S. algae* strain isolated from blood.

## 2. Materials and Methods

*S. algae* strain TYL was isolated on trypticase soy agar supplemented with 5% sheep blood (Becton Dickinson, San Jose, CA, USA) from the blood of a septic patient. The strain was identified by matrix-assisted laser desorption/ionization time-of-flight mass spectrometry (MALDI-TOF MS, bioMérieux, Marcy-l'Etoile, France) and 16S ribosomal RNA gene sequencing. In brief, a PCR amplicon of the 16S ribosomal RNA gene was obtained with primers B27F (5′-AGAGTTTGATCCTGGCTCAG-3′) and U1492R (5′-GGTTACCTTGTTACGACTT-3′) and subjected to Sanger sequencing. Sequences obtained were blasted against the bacterial 16S ribosomal RNA gene sequences in the GenBank database (http://www.ncbi.nlm.nih.gov/) using the BLASTn (optimized for MegaBLAST) algorithm. Antibacterial susceptibility testing was performed by the Vitek 2 system (bioMérieux, Marcy-l′Etoile, France) according to the manufacturer's instructions. The reference strains *Escherichia coli* ATCC 25922 and *Pseudomonas aeruginosa* ATCC 27853 were used as quality controls. The broth microdilution method was used to determine the minimum inhibitory concentration (MIC) of colistin.

Genomic DNA was extracted by using the QIAGEN Genomic-tip 100/G kit and the Genomic DNA Buffer Set (QIAGEN, Paisley, UK) then quantified by using the Qubit dsDNA HS Assay kit and the Qubit 2.0 fluorometer (Life 3 Technologies, Carlsbad, CA, USA). The indexed PCR-free library preparation was constructed using a multiplexed high-throughput sequencing TruSeq DNA Sample Preparation kit (Illumina, San Diego, CA, USA) following the standard protocol provided by the manufacturer with minor modification. Low-quality reads and bases were removed and trimmed using the Kmer-based tool DUK (http://duk.sourceforge.net/) and FASTQ Trimmer (https://github.com/agordon/fastx_toolkit), respectively. The reads were assembled using Velvet version 1.2.07 [9]. Wgsim 0.3.0 (https://github.com/lh3/wgsim) was then used to generate 1–3 kb simulated paired-end reads. The genome was assembled using the ALLPATHS-LG v. R46652 [10] and annotated using the Prokaryotic Genomes Automatic Annotation Pipeline (PGAAP) (https://www.ncbi.nlm.nih.gov/genome/annotation_prok/). Protein family analysis is performed by RPSBLAST v. 2.2.15 [11] against COGs (Clusters of Orthologous Groups of proteins) databases (*E*-value cutoff 0.001) (https://warwick.ac.uk/fac/sci/moac/people/students/peter_cock/python/rpsblast/). Homologs of the *S. algae* TYL genes were BLAST searched against the Integrated Microbial Genomes and Microbiomes database v.5.0 [12], the Virulence Factor Database [13], and the Comprehensive Antibiotic Resistance Database [14] to identify candidate virulence genes and antibiotic-resistant genes.

## 3. Results and Discussion

**Figure 1:**
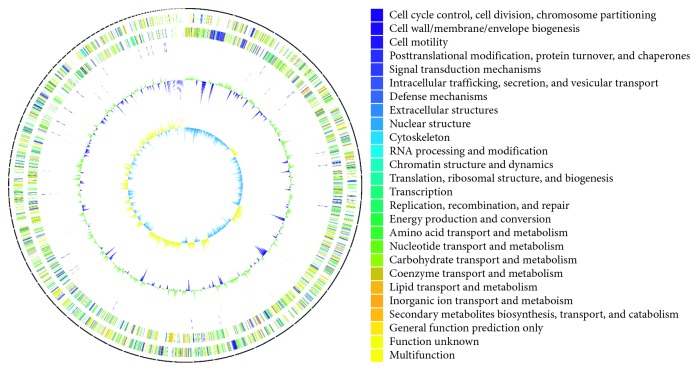
Circular genome map of *Shewanella algae* TYL. Circles from the outside to inside: (1) DNA coordinates; (2, 3) function-based color-coded mapping of the CDSs predicted on the forward and reverse strands. Functions are color-coded; (4) tRNA genes; (5) rRNA genes; (6) GC plot showing regions above the average (green) and below (violet); (7) GC skew showing regions above average (yellow) and below (light blue).

Whole-genome sequencing was performed using the Illumina MiSeq platform which generated 4,270,654 reads (mean read length of 301 bp) and a gross amount of 1,285,466,854 bp. The assembly of the draft genome sequence consists of 100 scaffolds amounting to 4,821,720 bp (total read depth of 267-fold coverage), and the *G* + *C* content is 52.95%. An illustration of the genomic contents in the genome of TYL is shown in Figure 1. The maximum contig size was 234,074 bp, and the N50 size was 96,168 bp. Of the 4,304 genes predicted, 4,112 were protein-coding genes, 13 RNAs, 90 tRNAs, and 6 noncoding RNAs. The distribution of genes into COGs functional categories is presented in Table 1. This Whole Genome Shotgun project has been deposited at DDBJ/ENA/GenBank under the accession number: LVDB01000000. The version described in this paper is version LVDB01000000.

**Figure 2:**
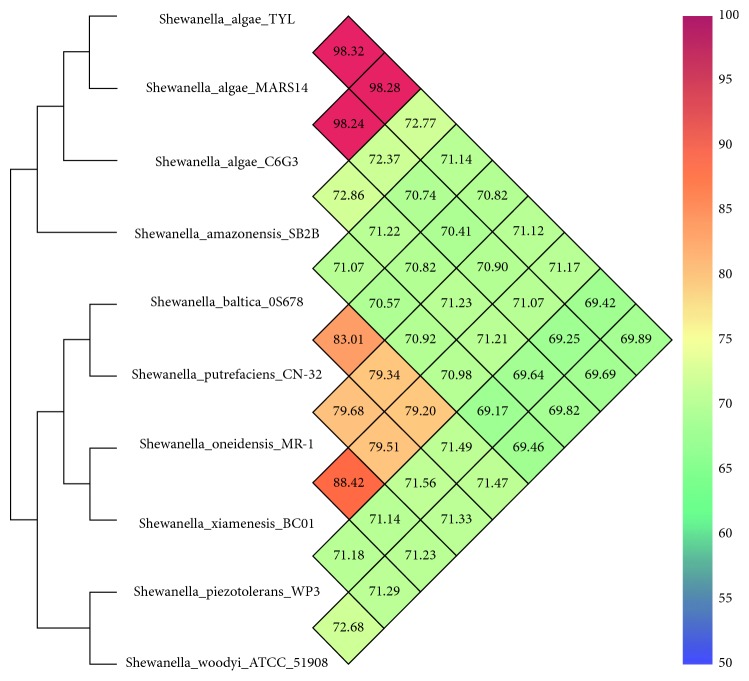
Heat-map and phylogenetic trees based on Average Nucleotide Identity values determined for *Shewanella algae* TYL and related strains. The values between two strains are given in the junction point of the diagonals departing from each strain. Figure two is reproduced from the study of Tseng et al. [16] (under the Creative Commons Attribution License/public domain).

To measure the nucleotide-level genomic similarity between TYL and the other *Shewanella* genomes, the Average Nucleotide Identity was calculated using a modified algorithm [15]. Results from this analysis showed an Average Nucleotide Identity (ANI) value higher than 98% with both the C6G3 (NCBI accession number: NZ_JPMA00000000.1) and MARS 14 strains (NCBI accession number: NZ_CDQH00000000.1), confirming the identification at the species level (Figure 2).

The TYL strain is resistant to colistin (MIC = 32 *μ*g/ml) and susceptible to imipenem (MIC ≤ 0.25 *μ*g/ml), piperacillin/tazobactam (MIC ≤ 4 *μ*g/ml), ceftriaxone (MIC ≤ 1 *μ*g/ml), ceftazidime (MIC ≤ 1 *μ*g/ml), cefepime (MIC ≤ 1 *μ*g/ml), gentamicin (MIC ≤ 1 *μ*g/ml), and amikacin (MIC ≤ 2 *μ*g/ml).

Over the past decade, the emergence of multidrug-resistant Gram-negative microorganisms increased the use of colistin as the remaining therapeutic option [17]. Nevertheless, the reports of colistin resistance globally are of great concern. The two most studied nonfermentative Gram-negative bacteria with acquired resistance to colistin are *Acinetobacter baumannii* and *Pseudomonas aeruginosa* [18]. Lipid A modification is a common mode of colistin resistance in clinical isolates belonging to these two species [19, 20]. We identified the homolog of *pmrC* gene associated with colistin resistance [21]. Analysis of the upstream and downstream of *pmrC* gene was performed. We found *pmrA* and *pmrE* genes located downstream of *pmrC* gene. We noted a similar arrangement in colistin-resistant *S. algae* MARS 14 (Figure S1). Functional genomic study on *S. algae* MARS 14 demonstrated that the colistin resistance in *S. algae* is associated with phosphoethanolamine transferase EptA-encoding *pmrC* gene [21]. EptA adds phosphoethanolamine to lipid A, resulting in structural modification of lipopolysaccharide and colistin resistance [21]. Studies of colistin resistance patterns of *S. algae* are limited to case series and case reports [22, 23]. It was suggested that all *S. algae* are resistant to colistin [24]. However, there are also reports of colistin-susceptible strains [25]. Large-scale comparative genomics focus on *pmr* genes, and colistin resistance phenotype are needed to fully exploit mechanisms of colistin resistance in *S. algae*.

The emergence of carbapenem-resistant *S. algae* is a growing concern worldwide. Carbapenemase producers are of major concern. In this study, we detected a chromosome-encoded *bla*_OXA-55_ *ß*-lactamase gene. OXA-type carbapenemases are members of class D *ß*-lactamases with carbapenem-hydrolysing activities. The mechanisms of carbapenem resistance in *S. algae* are suggested to be caused by beta-lactamase gene *bla*_OXA-55_ [23]. Various *bla*_OXA_-type genes have been reported in different *Shewanella* species [26]. In addition, there are increasing numbers of studies on horizontal gene transfer in *Shewanella* that demonstrate the organism is a potential reservoir of antimicrobial resistance [27, 28]. However, the OXA-55 *ß*-lactamase is also present in carbapenem-susceptible *S. algae* [29]. The resistance of carbapenems in *S. algae* may involve a combination of OXA-55 *ß*-lactamase and another mechanism. Routine surveillance and further investigation are needed for the resistance emergence.

*S. algae* causes diseases in both humans and marine animals. The organism has been reported to cause various diseases in shrimp, marine fish, and shellfish [4, 5, 30]. In humans, *S. algae* infections have been associated with seafood ingestion and water exposure [6]. Severe bacteremic illness complicated with human shewanellosis is not uncommon [31]. Pathogenicity test showed the LD_50_ of *S. algae* was 1.8 × 10^4^ CFU/mL for 20-day-old abalone postlarvae [4]. Hemolysis was demonstrated in the pathogenic strains of *S. algae* [30].

Hemolytic activity has long been suspected as a virulence factor of *S. algae* [32]. The *S. algae* TYL genome carries genes encoding hemolysin A (*hlyA*) and hemolysin III (*hly*III). Hemolysin A belongs to RTX pore-forming toxin *α*-hemolysin, which rearranges membrane permeability and causes cell lysis. Genomic analysis of *S. algae* TYL in this report supports the previous study, which demonstrated the hemolytic activity of environmental *S. algae* and thus suggested it as a possible virulence determinant [33].

Biliary tract infection is one of the major presentations and possible ports of entry in shewanellosis [34]. Bile salts possess antimicrobial activity, causing damage to lipids in the cell membrane and DNA. Hence, *S. algae* must be able to survive the deleterious action of bile salts. A previous study demonstrated bile resistance in *S. algae* [24]. In this study, we identified genes associated with bile tolerance (*htpB*, *exbBD*, and *galU*). The results support the earlier genomic study, suggesting a common mechanism of bile resistance in *Shewanella* [16].

## 4. Conclusions

In summary, multiple resistance and virulence determinants in *S. algae* TYL were identified by whole-genome sequencing. To the best of our knowledge, this report describes the first *S. algae* blood isolate. Our data provide the impetus for further research on the zoonotic potential of *S. algae*.

## Figures and Tables

**Table 1:** COG functional categories of *Shewanella algae* TYL genome.

COG class	Description	Count	%
D	Cell cycle control, cell division, chromosome partitioning	37	0.88
M	Cell wall/membrane/envelope biogenesis	223	5.16
N	Cell motility	69	1.73
O	Posttranslational modification, protein turnover, and chaperones	193	4.50
T	Signal transduction mechanisms	223	5.35
U	Intracellular trafficking, secretion, and vesicular transport	76	1.80
V	Defense mechanisms	91	2.18
W	Extracellular structures	0	0.00
A	RNA processing and modification	2	0.05
J	Translation, ribosomal structure, and biogenesis	193	4.64
K	Transcription	248	5.49
L	Replication, recombination, and repair	183	6.30
C	Energy production and conversion	277	6.61
E	Amino acid transport and metabolism	258	5.99
F	Nucleotide transport and metabolism	89	2.01
G	Carbohydrate transport and metabolism	109	2.60
H	Coenzyme transport and metabolism	135	3.20
I	Lipid transport and metabolism	111	2.51
P	Inorganic ion transport and metabolism	257	5.99
Q	Secondary metabolites biosynthesis, transport, and catabolism	52	1.23
R	General function prediction only	0	0.00
S	Function unknown	1,288	29.79
—	Multifunction	80	1.99

## Data Availability

This Whole Genome Shotgun project has been deposited at DDBJ/ENA/GenBank under the accession LVDB01000000. The version described in this paper is version LVDB01000000.

## Supplementary Materials

Supplementary Materials Figure S1: genetic context of pmrC in *Shewanella algae* MARS 14 and *Shewanella algae* TYL. The arrows indicate the positions and directions of transcription for each gene.
