# Supplementary material for: Genome Sequence of Colistin-Resistant Bacteremic Shewanella algae Carrying the Beta-Lactamase Gene bla OXA-55
Source: Can J Infect Dis Med Microbiol. 2019 Jun 10;2019:3840563. doi: 10.1155/2019/3840563 (PMC6590586; doi:10.1155/2019/3840563)
Supplement: Supplementary Materials — Figure S1: genetic context of pmrC in Shewanella algae MARS 14 and Shewanella algae TYL. The arrows indicate the positions and directions of transcription for each gene. [file 3840563.f1.docx]

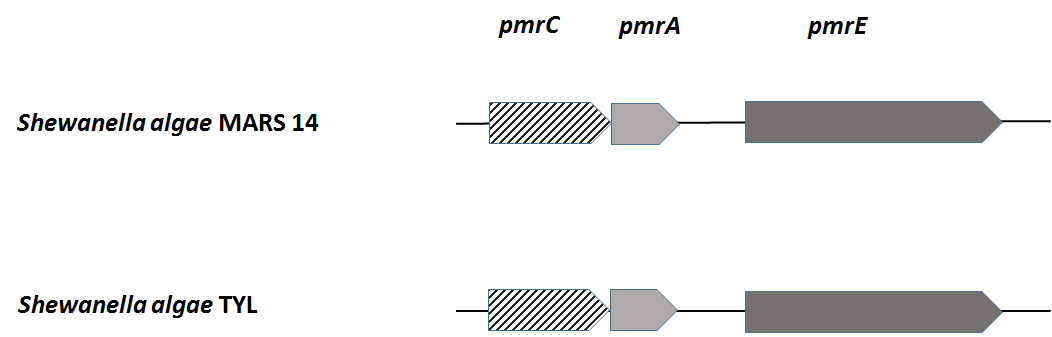


**Supplementary Material Figure S1.**

Genetic context of *pmrC* in *Shewanella algae* MARS 14 and *Shewanella algae* TYL. The arrows indicate the positions and directions of transcription for each gene.
